# The Chinese Herbal Formula Shenzhu Tiaopi Granule Results in Metabolic Improvement in Type 2 Diabetic Rats by Modulating the Gut Microbiota

**DOI:** 10.1155/2019/6976394

**Published:** 2019-06-04

**Authors:** Jindong Zhao, Yan Li, Min Sun, Ling Xin, Tianming Wang, Liangbing Wei, Chanjuan Yu, Mengmeng Liu, Yingqun Ni, Ruimin Lu, Taotao Bao, Lu Zhang, Yuanyuan Wu, Zhaohui Fang

**Affiliations:** ^1^Department of Endocrinology, The First Affiliated Hospital of Anhui University of Chinese Medicine, Hefei 230031, China; ^2^Diabetes Institute, Anhui Academy Chinese Medicine, Hefei 230012, China; ^3^Department of Infectious Disease, The First Affiliated Hospital of Anhui University of Chinese Medicine, Hefei 230031, China; ^4^School of Life Sciences, Anhui University, Hefei 230039, China; ^5^Department of Computer Center, The First Affiliated Hospital of Anhui University of Chinese Medicine, Hefei 230031, China; ^6^Department of Technology, Anhui University of Chinese Medicine, Hefei 230038, China; ^7^Department of Pharmacy, The First Affiliated Hospital of Anhui University of Chinese Medicine, Hefei 230031, China

## Abstract

**Objective:**

The aim of this study is to investigate the implication of the Chinese herbal formula (CHF) Shenzhu tiaopi Granule (STG) in type 2 diabetes mellitus (T2DM) and discuss the mechanisms by which STG regulates the gut microbiota.

**Method:**

Goto-Kakizaki (GK) rats and age-matched Wistar (W) rats were acclimatized for 1 week. The GK rats were randomly divided into 3 groups and orally gavaged with saline (model group, M), acarbose (acarbose group, A), and STG (granule of CHF group, G; the component of this formula includes* Codonopsis pilosula*,* Rhizoma Atractylodis*,* Pinellia*,* Poria cocos*,* Pericarpium Citri Reticulatae*,* Coptis chinensis Franch*,* and Pueraria*). The W rats were orally gavaged with saline (control group, C). The observation time was 8 weeks. The weight, fasting blood glucose (FBG) level, and blood lipid levels were tested. The 16S rRNA genes in the V3-V4 region were sequenced, and the structure of the gut microbiota was analysed.

**Results:**

Compared to C, M displayed significant differences in blood glucose, gut microbiota, etc. (P<0.05; P<0.01). Compared to M, A and G showed a similar reduction in the FBG gain and a shift in the structure of the gut microbiota (P<0.05; P<0.01). Compared with A, G exhibited a significant decrease in weight, FBG level, and total cholesterol (P<0.05). The gut microbiota, Bacteroidetes, the Firmicutes/Bacteroidetes ratio,* Allobaculum*, and Desulfovibrionaceae were significantly decreased in response to the STG treatment, while* Lactobacillus* was significantly enriched (P<0.05; P<0.01). The community composition also differed at the phylum and genus levels based on the linear discriminant analysis effect size and heat map.

**Conclusion:**

Our findings suggest that the composition of the gut microbiota was significantly changed in the diabetic GK rats compared with that in the normal W rats. STG treatment can improve glucose and lipid levels and modulate the gut microbiota in T2DM rats.

## 1. Introduction

Recently, some scholars have suggested that the gut microbiota is the second genome of the human body. The composition and balance of the gut microbiota have been reported to play a critical role in human health [1]. Our knowledge of gut microbiota function has increased. The gut microbiota can contribute to the onset of metabolic and energy metabolic dysregulation [2, 3]. Type 2 diabetes mellitus (T2DM) has been shown to be associated with the establishment and maintenance of an imbalance in the microbiota; this imbalance is primarily characterized by an increased abundance of* Enterococcus*,* Enterobacter*, and Betaproteobacteria and a decreased abundance of* Bifidobacterium*,* Lactobacillus*, class Clostridia, and* Faecalibacterium prausnitzii* in animal studies [4–7].

Many studies have shown that the gut microbiota modulates glucose homoeostasis following treatment with metformin, acarbose, GLP-1, vildagliptin, SGL-2, etc. [8–14]. Acarbose inhibits alpha glucosidase in the proximal small intestine. Its mechanism of action is consistent with that of a Chinese diet, and, thus, acarbose is widely used in China [15]. Benli SU [10] found that acarbose treatment increased the abundance of gut* Bifidobacterium longum* in Chinese patients with T2DM. These studies demonstrated that the modulation of the gut microbiota contributes to acarbose-induced decreases in blood glucose.

For many years, Chinese herbal formulas (CHFs) have been used to treat many diseases, such as diabetes. Several studies have shown that CHF effectively reduces the blood glucose and glycated haemoglobin A1c (HbA1c) levels in T2DM and prediabetic patients [16–19]. In our previous research, Shenzhu tiaopi Granule (STG), which is a CHF, was tested in prediabetic and T2DM patients. STG was shown to prevent insulin resistance and decrease HbA1c activity [19]. However, whether the gut microbiota composition is involved in STG-mediated blood glucose regulation is still unclear. Therefore, in this study, the possible metabolic improvement and gut microbiota modulatory activities of the CHF STG were investigated.

## 2. Methods and Materials

### 2.1. Animal Experiments

All animal procedures were performed in accordance with the principles approved by the Animal Ethics Committee of Anhui Chinese Medicine University. Thirty male Goto-Kakizaki (GK) rats (8 weeks old) and six age-matched Wistar (W) rats were purchased from the Shanghai Laboratory Animal Center (Shanghai, China, certificate number 2015000531632) and housed at a controlled temperature from 21°C to 26°C with from 40% to 50% relative humidity. The animals were housed in a specific pathogen-free animal laboratory and maintained on a 12 h light-dark cycle with ad libitum access to standard diet and water. All rats were fed a pulverized standard rat pellet diet. Following acclimatization for 1 week, GK rats with high fasting blood glucose (FBG) levels exceeding 11.1 mmol/L were used for the subsequent experiments. The experimental GK rats were randomly divided into the following 3 groups: the model group (M, T2DM, n=6), which received saline intragastrically once daily; the acarbose group (A, n=6), which received 50 mg/kg body weight acarbose intragastrically once daily (Bayer Health Care Co., Ltd.); and the STG CHF group (G, n=18), which received 1000 mg/kg body weight STG intragastrically once daily. The W rats were used as the control group (C, n=6) and were intragastrically treated as the model group. No rats died during the experiment.

### 2.2. Sample Collection and Measurement

After 8 weeks, the animals were anaesthetized with sodium pentobarbital (60 mg/kg), and blood samples were collected via abdominal aorta puncture sampling to obtain isolated serum by centrifugation at 4000 rpm for 10 min. Then, the colon content was collected.

The body weight was measured every week. The FBG, triglyceride (TG), total cholesterol (TC), high-density lipoprotein cholesterol (HDL-C), and low-density lipoprotein cholesterol (LDL-C) levels in the rats were determined by an automatic biochemical analyser (Olympus AU640).

### 2.3. Illumina MiSeq Pyrosequencing of the Gut Microbiota

Microbiota genomic DNA was extracted from colon faecal samples from six rats per group by using Fast DNA SPIN extraction kits (MP Biomedicals, Santa Ana, CA, USA) following the manufacturer's instructions and stored at -20°C prior to further analysis. The quantity and quality of the extracted DNA were quantified by 2% agarose gel electrophoresis and a Nanodrop ND-1000 spectrophotometer (Thermo Fisher Scientific, NC2000, Waltham, United States). The microbiota V3-V4 regions of the 16S ribosomal RNA (rRNA) genes were amplified by PCR. The 338F/806R primer sequences used in the subsequent experiments were as follows: 5'-ACTCCTACGGGAGGCAGCA -3' and reverse: 5'-GGACTACHVGGGTWTCTAAT-3'. The PCR conditions were as follows: initial denaturation at 98°C for 2 min, followed by 25 cycles of denaturation at 98°C for 15 s, annealing at 55°C for 30 s, extension at 72°C for 30 s, and a final extension at 72°C for 5 min. The PCR amplicons were purified with Agencourt AMPure Beads (Beckman Coulter, Indianapolis, IN) and quantified using a PicoGreen dsDNA Assay Kit (Invitrogen, Carlsbad, CA, USA). After the individual quantification step, the amplicons were pooled in equal amounts, and pair-end 2×300 bp sequencing was performed using an Illumina MiSeq 2000 platform with MiSeq Reagent Kit v3 at Shanghai Personal Biotechnology Co., Ltd. (Shanghai, China).

All sequences were submitted to GenBank under accession number SRP091598. Sequences with mean Phred scores lower than 20, sequences containing ambiguous bases, and sequences with lengths shorter than 150 bp were removed. The paired-end reads were assembled using FLASH [20]. Effective sequence overlaps longer than 10 bp without mismatch in the primers were assembled according to their overlap sequence. The OTU taxonomic classification was conducted by BLAST searching the representative sequences against the Greengene Database [21] using the best hit [22]. The trimmed sequences were uploaded to Quantitative Insights into Microbial Ecology (QIIME, v1.8.0) for further analysis.

### 2.4. Statistics and Analysis

All data are expressed as the mean ± standard error of the mean and were subjected to a one-way analysis of variance (ANOVA) or Fisher's Least Significant Differences Test (LSD) using SPSS 20.0 (SPSS Inc., Chicago, IL, USA). A P-value<0.05 was considered indicative of statistical significance.

The sequences were analysed with the QIIME and R packages (v3.2.0) [23, 24]. A Venn diagram was generated to visually represent the number of shared and unique OTUs among the groups. The alpha diversity indexes are represented according to the Shannon index [25]. The taxonomy compositions and abundances were visualized based on GraPhlAn or linear discriminant analysis (LDA) effect size (LEfSe) by the galaxy online analysis platform (http://huttenhower.sph.harvard.edu/galaxy/) [26]. The normalized abundance is shown in a heatmap generated using pheatmap.

The data were visualized via a principal coordinate analysis (PCoA) and unweighted pair-group method with arithmetic means (UPGMA) hierarchical clustering [27]. The significance of the differentiation of the microbiota structure among the groups was assessed by a permutational multivariate analysis of variance (PERMANOVA) [28] and an analysis of similarities (ANOSIM) [29, 30] using the R package.

## 3. Results

### 3.1. Metabolic Biomarker Levels after Treatment

**Figure 1:**
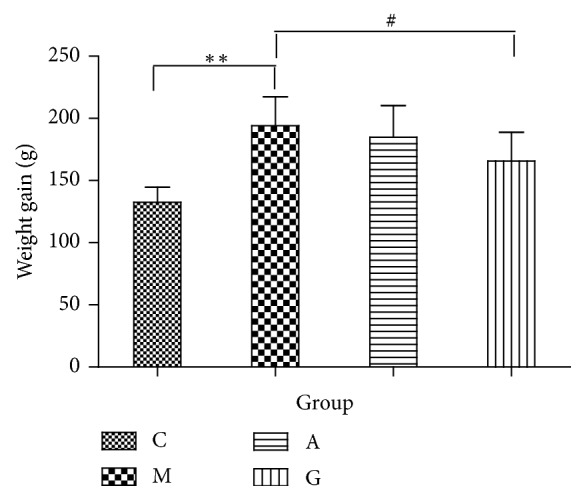
Comparison of body weights among the different groups. ^*∗∗*^P<0.01 vs the C group; ^#^P<0.05 vs the M group.

**Figure 2:**
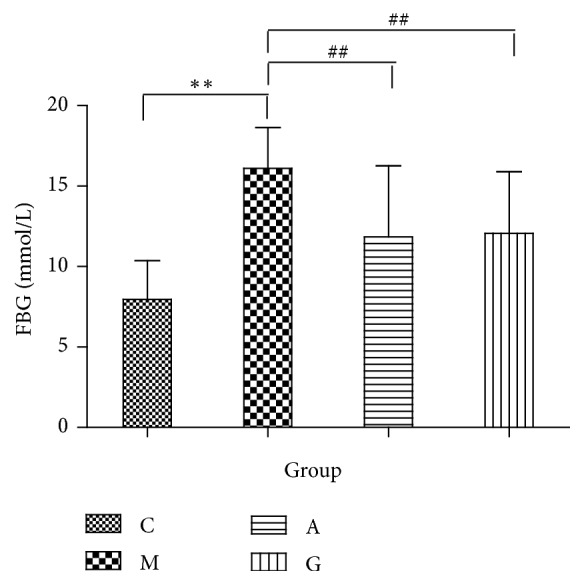
Comparison of the FBG levels among the different groups. ^*∗∗*^P<0.01 vs the C group; ^##^P<0.01 vs the M group.

**Figure 3:**
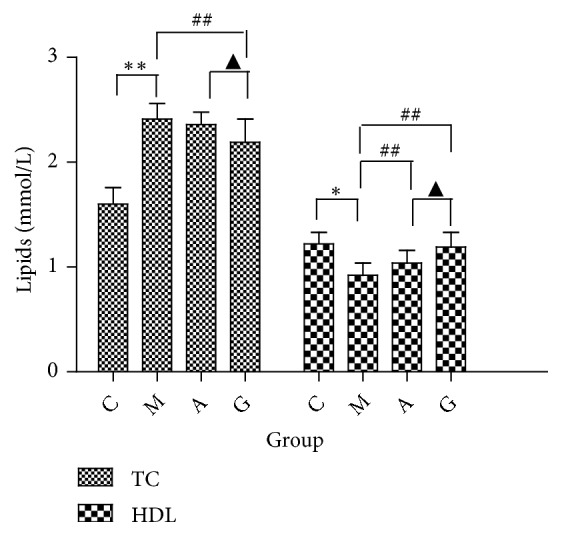
Comparison of the TC and HDL levels among the different groups. ^*∗*^P<0.05, ^*∗∗*^P<0.01 vs the C group; ^##^P<0.01 vs the M group; ^▲^P<0.05 vs the A group.

Before the experiment, the body weights did not significantly differ among the groups. From the beginning to the end of the experiment, the body weight gain was significantly increased in the M group compared with that in the C group. Compared with the M group, the G group showed a significantly reduced weight gain (P<0.05). The weight gain in the rats in the M and G groups did not significantly differ from that in the rats in the A group (Figure 1). The FBG levels did not significantly differ among the groups before the experiment. From the beginning to the end of the experiment, the FBG levels were significantly increased in the M group compared with those in the C group. Significant differences were observed in the FBG levels between the G and A groups and the M group. Meanwhile, compared with the A group, the G group did not show significantly reduced FBG levels (P<0.05) (Figure 2). The serum levels of TC and HDL were significantly elevated in both the M and C groups (P<0.01). However, no differences were observed in the LDL and TG levels. Compared with the M group, the A group showed significantly reduced levels of HDL (P<0.01). Compared with the M group, the G group had significantly reduced levels of TC and HDL (P<0.01). Meanwhile, compared with the A group, the G group showed significantly reduced levels of TC and HDL (P<0.05) (Figure 3).

### 3.2. Community Structure of the Gut Microbiota

**Figure 4:**
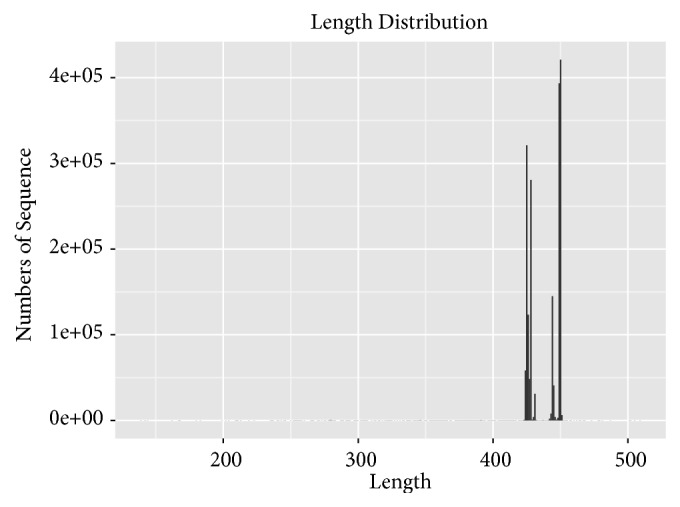
Sequence length distribution map.

**Figure 5:**
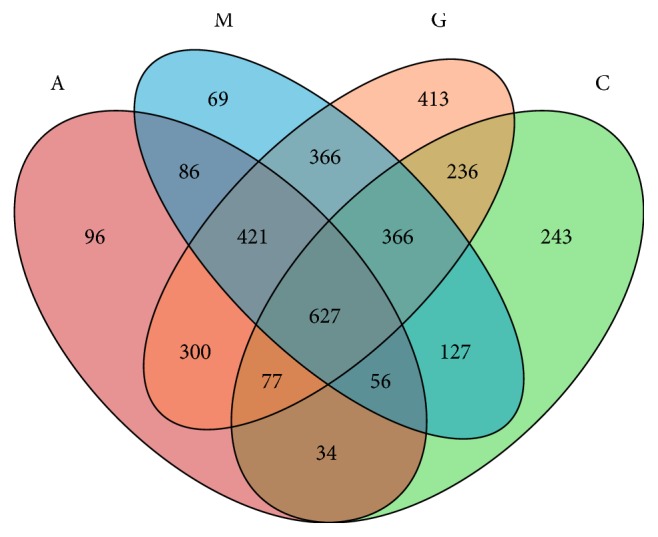
Venn diagram of the common and unique OTUs in the four groups.

To assess the changes in the gut microbiota community, 16S rRNA gene sequencing of variable regions V4-V5 of bacteria from the four groups was performed using Illumina MiSeq platforms. We produced a total of 2,132,752 sequences with an average of 49,219 sequences per C group sample, 51,127 sequences per M group sample, 50,763 sequences per A group sample and 68,116 sequences per G group sample. Thirty-six faecal sample sequences had an average length of 420~460 base pairs (Figure 4). The results are presented as operational taxonomic units (OTUs). The data were binned with a homology cut-off value of 97% against the Greengene database. In total, 15 qualified sequences (>0.001%) were clustered into 3,517 bacterial OTUs, and 243 OTUs were found only in the C group, 69 OTUs were found only in the M group, 96 OTUs were found only in the A group, and 413 OTUs were specifically identified in the G group (Figure 5).

**Figure 6:**
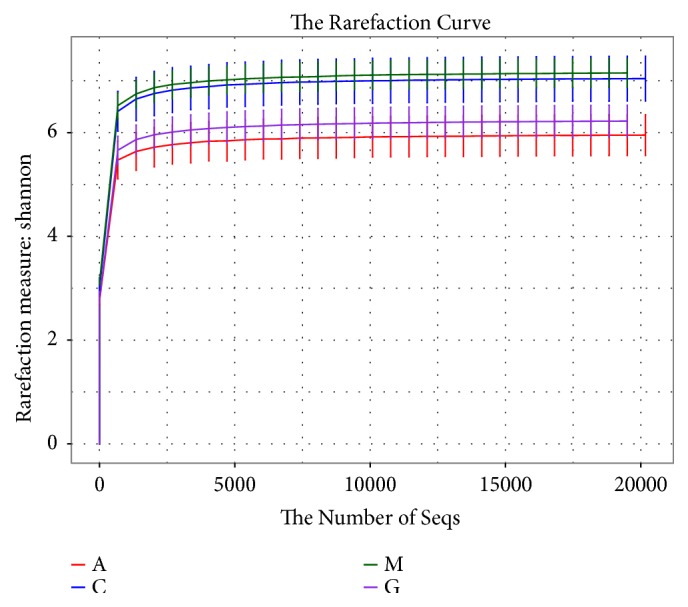
Shannon curve analysis of the different groups at the OTU level.

**Figure 7:**
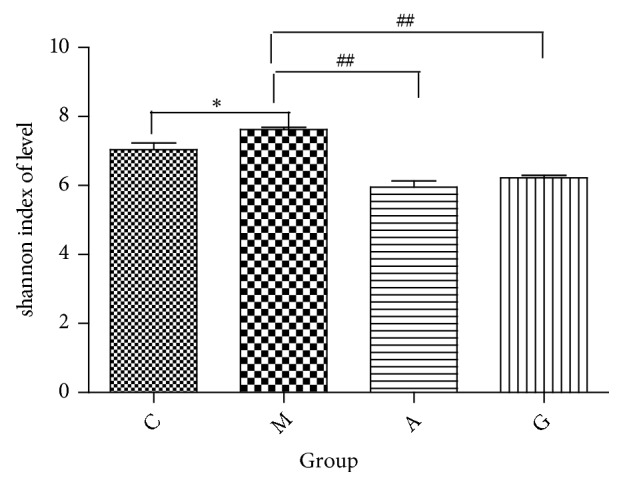
Shannon index analysis of the different groups at the OTU level. ^*∗*^P<0.05 vs the C group; ^##^P<0.01 vs the M group.

After rarefication, the sequencing depth of all samples was calculated as the current sequencing depth. Shannon curves covered rare new phenotypes and most of the diversity (Figure 6). At the OTU level, the community richness as measured by the Shannon index was significantly increased in the M group compared with that in the C group (P<0.001). Significant differences were observed between the A and the G groups and the M group (P<0.01). No significant differences in Shannon diversity were observed following treatment in the A and G groups (P<0.01) (Figure 7).

**Figure 8:**
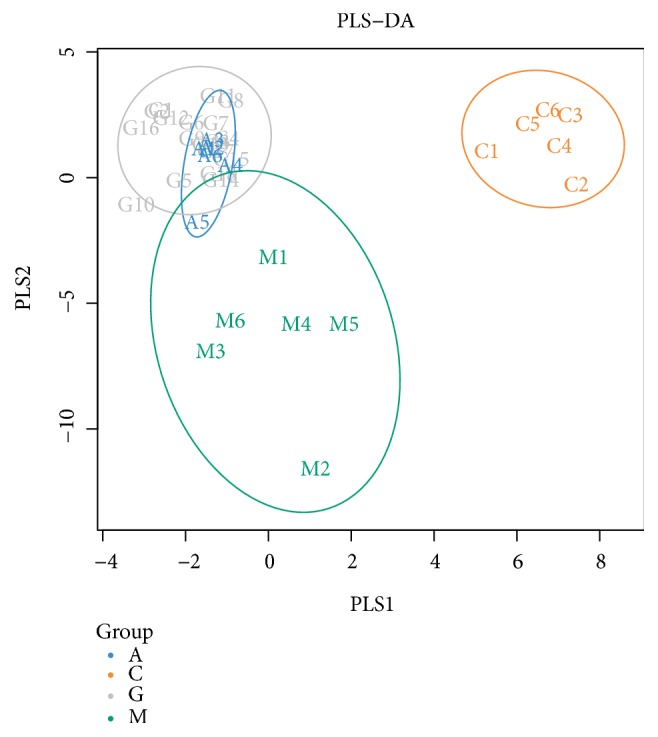
Partial least squares discriminant analysis chart.

**Figure 9:**
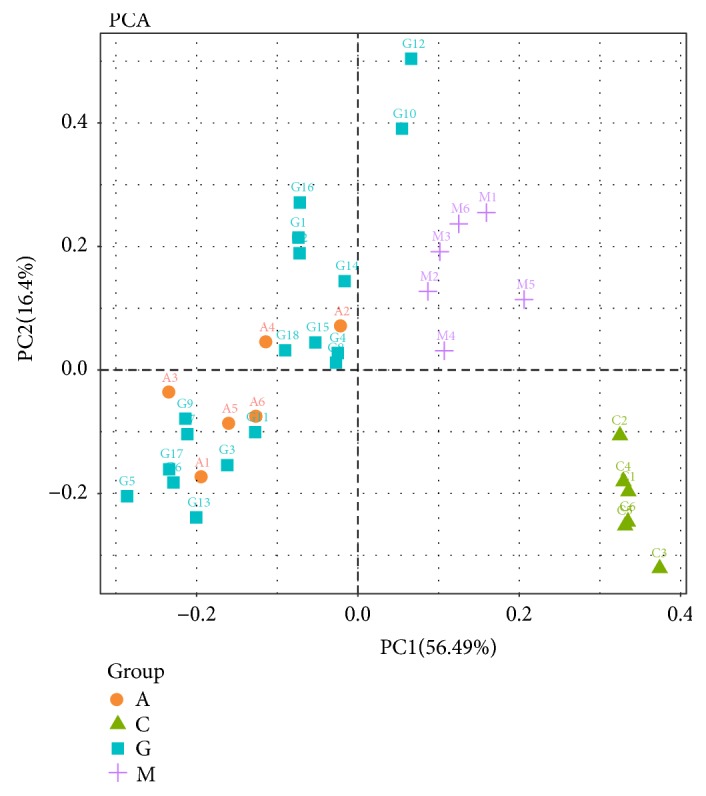
Principal coordination analysis score of the gut microbiota among the four groups.

**Figure 10:**
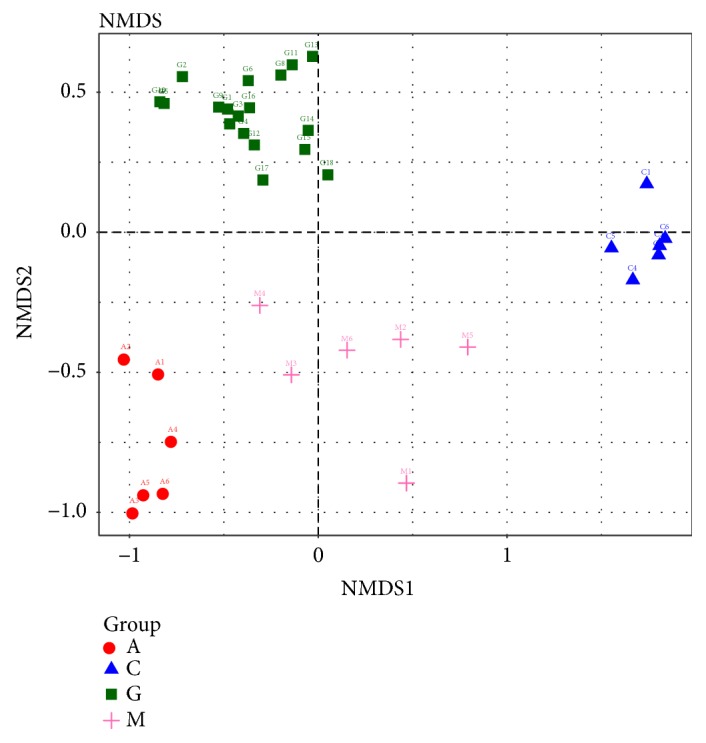
Nonmetric multidimensional scaling score based on the unweighted UniFrac distances of the microbiota.

Subsequently, a supervised PLS-DA model was constructed in which the M group was separated from the C group by PLS1, while the G and A groups were separated from the M group by PC2 (Figure 8). Unsupervised multivariate statistical methods, including PCoA score plots, were used to compare the distinct differences in the OTU abundance among the M and C groups by QIIME. Each point in the plot represents the gut microbiota of one sample. Furthermore, we observed that the treatments applied in the G and A groups altered the bacterial structure of the mouse intestine as evaluated by PCoA. The distance between the G groups was greater than that between the A groups. However, the gut microbiota structure of the G and A groups showed the same trend (Figure 9). The unweighted UniFrac-based nonmetric multidimensional scaling (NMDS) revealed a distinct clustering of the microbiota composition in each group among the four groups. The G groups exhibited a distinct microbiota composition that clustered differently from that of the other three groups (Figure 10).

### 3.3. Modulation of the Key Community of Gut Microbiota after Treatment

**Figure 11:**
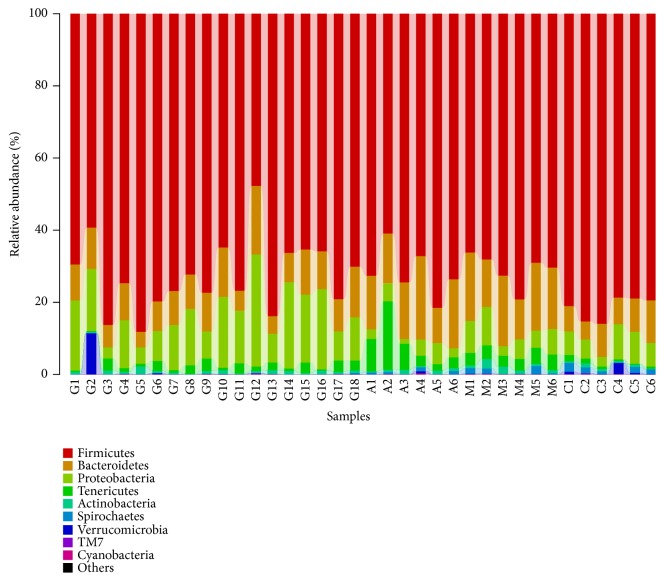
Composition abundance of gut microbiota at the phylum level.

**Figure 12:**
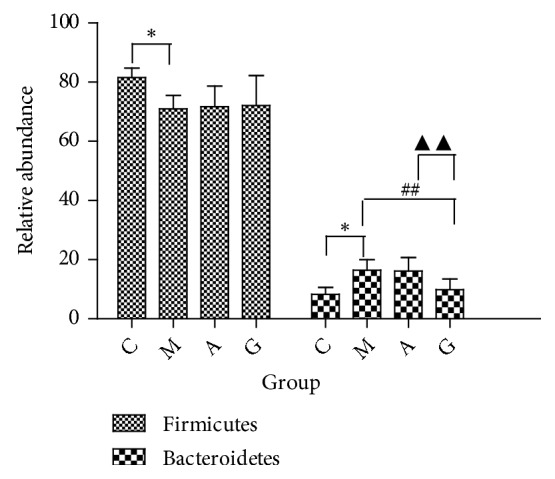
Relative abundance of bacterial phyla detected in faecal samples. ^*∗*^P<0.05 vs the C group; ^##^P<0.01 vs the M group; ^▲▲^P<0.01 vs the A group.

**Figure 13:**
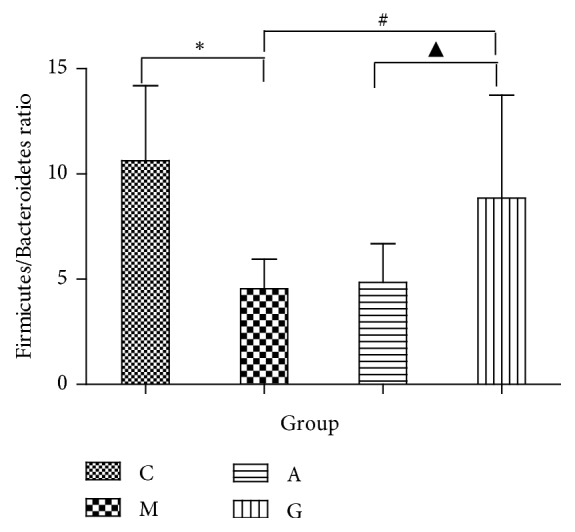
Firmicutes/Bacteroidetes ratio in the four groups. ^*∗*^P<0.05 vs the C group; ^#^P<0.05 vs the M group; ^▲^P<0.05 vs the A group.

The major phyla found in the gut microbiota diversity analysis included Firmicutes, Bacteroidetes, Proteobacteria, Tenericutes, and Actinobacteria, contributing 73.6%, 11.7%, 10.0%, 2.8%, and 0.8%, respectively, of all OTUs. Firmicutes was the most abundant phylum in all samples, accounting for 72.5% (in the G group), 71.7% (in the A group), 70.9% (in the M group), and 81.6% (in the C group) (Figure 11). An increase in the relative abundance of Bacteroidetes and a decrease in Firmicutes were detected in the M group compared to the C group (P<0.01). However, compared with the M group, the G and A groups showed no significant enrichment in the relative abundance of Firmicutes (P>0.05). Meanwhile, there were no significant differences between the G and the A groups (P>0.05). Compared with the M group, the G group showed a significantly enriched relative abundance of Bacteroidetes (P<0.05). Meanwhile, there were significant differences between the G and M groups and the A group (P<0.05). However, there were no observed differences in the A and M groups (Figure 12). The gut microbiota in the M group was characterized by a decreased proportion of Firmicutes/Bacteroidetes compared with that in the C group. The ratio showed significant differences, and higher abundances were observed in the G group compared with those in the M and A groups (P<0.05). Furthermore, there were no significant differences between the M and the A groups (P>0.05) (Figure 13).

**Figure 14:**
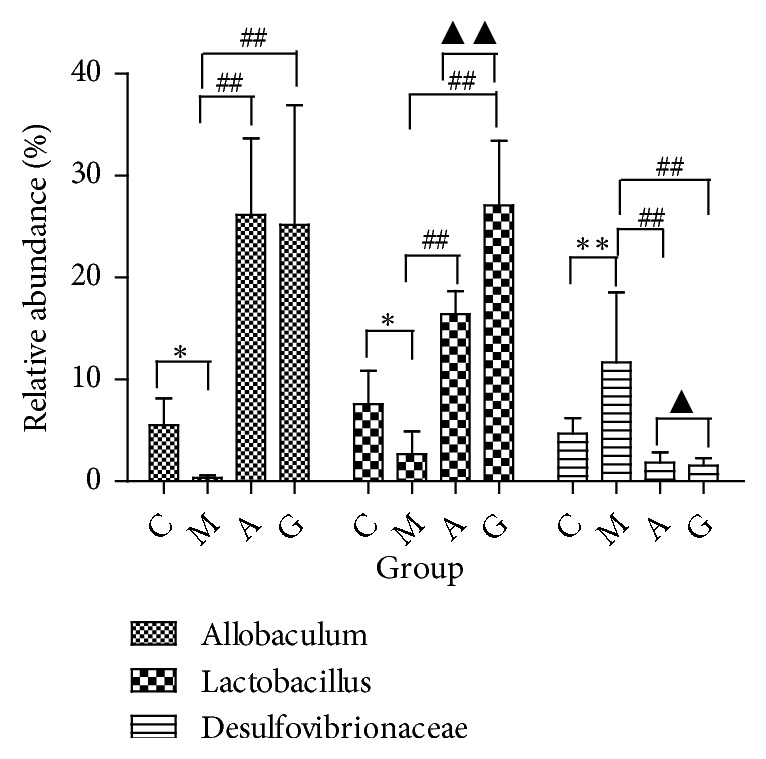
Relative abundance of genera detected in faecal contents. ^*∗*^P<0.05, ^*∗∗*^P<0.01 vs the C group; ^#^P<0.05 vs the M group; ^▲^P<0.05 vs the A group.

At the genus level, the microbiota composition greatly varied among the four groups. The relative abundances of* Allobaculum*, Clostridiales, Ruminococcaceae,* Lactobacillus*, S24-7, and Desulfovibrionaceae contributed 17.8%, 16.1%, 11.9%, 10.1%, 9.9%, and 7.1% of the total faecal microbiota population, respectively. A reduction in* Allobaculum* and* Lactobacillus* was detected in the M group compared with those in the C group. Compared with the M group, the A and G groups showed significantly increased abundances (P<0.05). A higher abundance of* Lactobacillus *was observed in the G group compared with that in the A group (P<0.01). An increase in Desulfovibrionaceae was detected in the M group compared with that in the C group. Compared with the M group, the A and G groups showed significantly decreased levels (P<0.05). No lower abundance of Desulfovibrionaceae was observed in the G group compared with that in the A group (P<0.01) (Figure 14).

**Figure 15:**
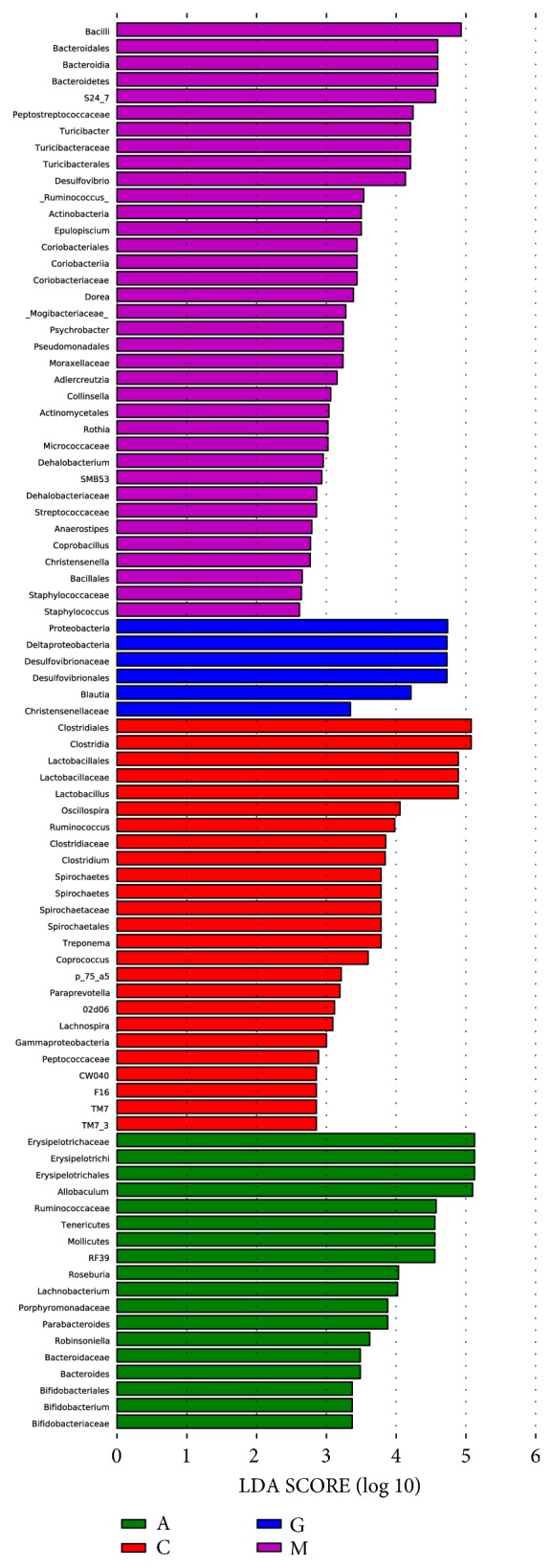
LEfSe comparison of gut microbiota among the four groups.

**Figure 16:**
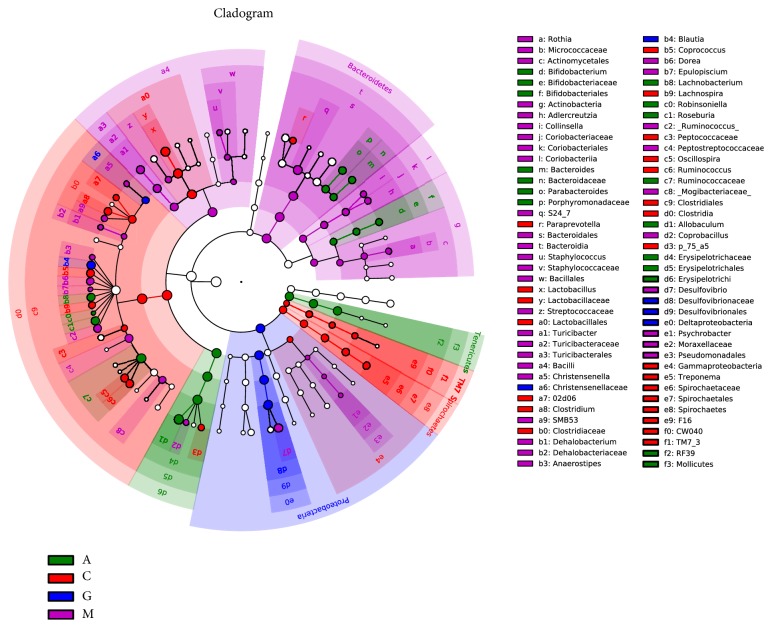
Taxonomic cladogram derived from the LEfSe analysis of the four groups. Yellow nodes represent no significant intergroup differences. Red and green nodes indicate significant intergroup differences.

**Figure 17:**
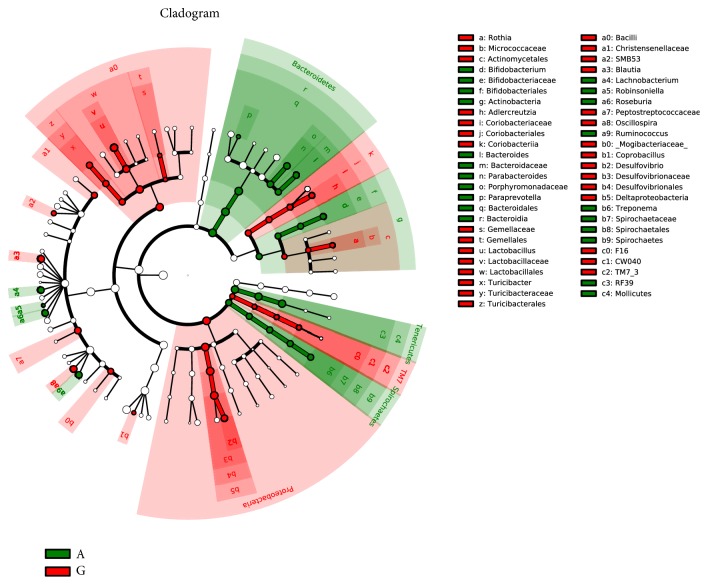
Taxonomic cladogram derived from LEfSe analysis comparing the A and G groups. Yellow nodes represent no significant intergroup differences. Red and green nodes indicate intergroup differences.

The LEfSe method was used to determine the differentially relatively abundant microbiota taxa and organisms with significant differences in gene abundance among the four groups. The length of the histogram indicates the extent of the influence of different organisms. The alpha value of the nonparametric factorial Kruskal-Wallis rank sum test reached the significant level of <0.05, and the threshold of the logarithmic LDA score for an increased relative abundance was >2.0. The relative abundance of the significantly different species was determined based on the LEfSe results (P<0.05) (Figure 15). The circles radiating from inside to outside represent the classification level from phylum to genus. The cladogram revealed that 12, 8, and 9 taxa were decreased, while 40, 36, and 48 taxa were increased in the C, A, and G groups, respectively, compared with those in the M group (Figure 16). Furthermore, 32 main genera were increased, while 24 main genera were decreased in the G group compared with those in the A group (Figure 17). Most of these genera originated from Proteobacteria, Actinobacteria, Verrucomicrobia, and Clostridia.

**Figure 18:**
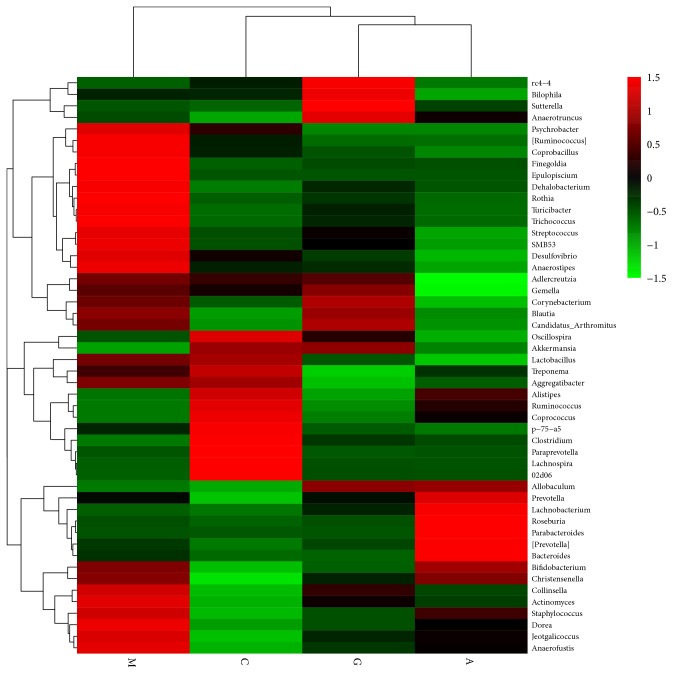
Heat map of faeces at the genus level. Red and green colours indicate high and low values of the percent of reads classified at that rank.

For further identification of the faecal microbiota community changes in the diabetes rat model, 50 genera with relative abundances were presented by a heat map and clustered at the genus level. Apparently, the genera were observed at different levels in the four groups.* Psychrobacter*,* Coprobacillus*,* Finegoldia*,* Epulopisclum*,* Dehalcbacterium*,* Rothia*,* Turicibacter*,* Trichococcus*,* Streptococcus*,* Smb53*,* Desulfovibrio*,* Anaerostipes*,* Collinsella*,* Actinomyces*,* Staphylococcus*,* Dorea*,* Jeotgalicoccus*, and* Anaerofustis* were increased in the M group, while the relative abundances of* Akkermansia*,* Alistipes*,* Ruminococcus*,* Coprococcus*,* Clostridium*,* Paraprevotella*, and* Lachnospira *were decreased (Table 1). The A and G groups exhibited the reverse of the changes in these species. Thus, the heat map of the relative abundance of microbiota species altered by the treatment in the G group shows the differences in the gut bacterial compositions compared to those in the A group. The heat map shows that the G group was more closely related to the C group than the A group (Figure 18).

## 4. Discussion

T2DM is caused by various lifestyle and environmental factors, including lack of exercise, stress, diet, and genetic factors. GK rats represent a model widely used to study T2DM [31], which is a progressive metabolic disease characterized by hyperglycaemia and dyslipidaemia [32]. Our findings demonstrated that, in GK rats, the body weight gain and FBG and TC levels were higher than those in normal W rats. However, the HDL level was lower than that in normal rats. In a previous study, the gut microbiota composition was shown to play a major role in diabetes [33]. In our study, the microbiota community in the C group varied considerably from that in the M group. Our study demonstrated a significant increase in gut microbiota diversity in the GK rats compared with that in the W rats (P<0.05). Compared to the C group, the M group showed a significantly increased Shannon index, Bacteroidetes abundance, and serum LDL-C level and significantly decreased Firmicutes abundance and Firmicutes/Bacteroidetes ratio. Regarding whether Firmicutes increased or decreased, the results are inconsistent, and the underlying mechanisms are not understood. Larsen N [4] and Qin J [5] reported that Firmicutes decreased in T2DM. However, Wei X [34] and Zhao L [35] reported an increase in this parameter. The bacterial flora in the M group was separated from that in the C group by the PLS-DA model. The relative abundances of various genera are shown at different levels in the M and C groups by heat maps and clustering.

Various therapeutic tools, such as lifestyle changes, pharmacological agents, CHF, and several natural polyphenols, are used to control diabetes [36]. In this experiment, acarbose and STG were selected for a comparative study. STG is a CHF. The efficacy of STG as a glycaemic control agent in the blood has been confirmed [19]. The natural compounds contain 7 medicinal components, namely,* Codonopsis pilosula*,* Rhizoma Atractylodis*,* Pinellia*,* Poria cocos*,* Pericarpium Citri Reticulatae*,* Coptis chinensis Franch*, and* Pueraria*, at a ratio of 5:5:4:4:5:1:10. The CHF was authenticated and standardized based on marker compounds according to the Chinese Pharmacopoeia 2005 [37]. Jing Yp indicated that* Codonopsis pilosula* polysaccharides have good prebiotic properties by inhibiting the growth of* Desulfovibrio*,* Alistipes*, and* Helicobacter* and stimulating the growth of* Bifidobacterium*,* Lactobacillus*, and* Akkermansia* [38]. Wang JH showed that* R. Atractylodis* significantly enhances the abundance of* Bifidobacterium* and* Akkermansia* and the Bacteroidetes/Firmicutes ratio [39]. Berberine is a major pharmacological component of* C. chinensis* Franch that can reduce gut microbiota diversity and promote the gut microbiota to produce short-chain fatty acids, further contributing to its biological effects [40, 41]. Pueraria is a dietary flavone resistant to degradation that can change energy metabolic degradation pathways in the gut microbiota [42–44]. Our findings are consistent with these results and reveal significant gut microbiota alterations in T2DM in response to Xiexin Tang treatment [34].

Our current results show that the hypoglycaemic effect and elevated levels of HDL markedly improved with acarbose. Acarbose is a powerful drug in T2DM. The G group showed a similar trend. Additionally, the fasting glucose level in the A and G groups was significantly lower than that in the M group. The HDL levels were significantly higher than those in the M group. Meanwhile, the G group showed the best reduction in weight when compared with the M group and reduced TC when compared with the A group. We hypothesized that the hypolipidaemic effect may be attributed to variations in bile acid metabolism. We did not observe statistically significant changes in the FBG level following the acarbose or STG treatment, which may be due to the short intervention course. Therefore, we need to perform these tests with a prolonged duration.

The results also suggest that the gut microbiota in the rats with T2DM was substantially changed in the A and G groups compared to that in the M group, but there were also some significant differences with clinically meaningful benefits. The effects of microbiota dysbiosis can be significantly modulated by treatment. The present study assessed the correlations among the bacterial OTUs. The bacterial diversity was decreased in the G and A groups. The alpha diversity indicated significant decreases in bacterial diversity in the G and A groups. The increased Shannon index was improved in the A and G groups (P<0.01), but there was no difference between the two groups.

The effects of acarbose and STG on bacterial diversity were consistent based on the PCoA score plot, unweighted beta diversities, and UniFrac distances. Therefore, the acarbose and STG treatments may decrease the overall bacterial diversity, and the specific composition of the microbiota was strongly associated with metabolic improvement following treatment.

There were no significant differences in the increasing abundance of Firmicutes in the G, A, and M groups. STG changed the structure of the gut microbiota community, especially by reducing the abundance of Bacteroidetes compared to that in the M and A groups (P<0.01). The gut microbiota in T2DM is more abundant and exhibits increased Bacteroidetes levels compared to that in controls with normal glucose metabolism [5, 45], which might explain the lack of significant differences in food intake, but the weight gain in the G group was lower than that in the M group (P<0.05). The ratio of Firmicutes/Bacteroidetes was elevated in the M group, which is consistent with the results reported in many previous studies [4], and was higher in the G group compared with that in the M and A groups (P<0.05). However, some studies have shown that the ratio of Firmicutes/Bacteroidetes is decreased following treatment [5, 34]. The specific cause of the increased Firmicutes/Bacteroidetes ratio in the G group is unclear.

In our current study, acarbose and STG increased the relative abundances of* Allobaculum* and* Lactobacillus* in the phylum Firmicutes and decreased Desulfovibrionaceae in the phylum Proteobacteria. Notably, we found that compared with the A group, the G group showed a selective increase in* Lactobacillus *and a decrease in Desulfovibrionaceae. In a previous study,* Lactobacillus* was reported to be negatively correlated with IGT and T2DM, while Desulfovibrionaceae was positively correlated with these factors [46, 47].* Lactobacillus *enhances insulin secretion in T2DM and modifies the structure of polysaccharides to regulate the gut microbiota [48, 49]. Furthermore, a high proportion of* Lactobacillus* is associated with reduced levels of TC [35, 50]. Moreover, Desulfovibrionaceae, which is an important endotoxin producer, can damage the gut barrier [51, 52]. Our analysis revealed that* Lactobacillus* and Desulfovibrionaceae were significantly associated with T2DM. Therefore, diabetes was effectively controlled in the G group by the STG supplementation.

In this study, the LEfSe analysis identified potential biomarkers in the A and G groups related to their gut microbiota. Acarbose selectively increased species in the phylum Firmicutes. However, STG increased Proteobacteria. In a previous study, the abundance of Proteobacteria was reported to be negatively correlated with T2DM [45, 53]. We found increased Deltaproteobacteria, Desulfovibrionales, and Desulfovibrionaceae, which could explain the improved hyperglycaemia.

In this study, the 16S rRNA gene sequencing analysis suggested that enrichment in the gut bacteria at the genus level significantly differed between the A and the G groups; these changes included* Akkermansia*,* Adlercreutzia*,* Bacteroides*,* Roseburia*,* Parabacteroides*, and* Dorea*. The increased relative abundance of* Akkermansia* could prevent T2DM [54, 55]. We found that STG also increased the abundance of* Akkermansia*. Increased* Adlercreutzia* has been detected in chronic kidney disease [56] and may be a risk factor for the development of diabetic kidney disease. We found that STG can decrease the abundance of* Adlercreutzia*.

In summary, these findings suggest that gut microbiota modulation by STG might be involved in glycometabolism and lipometabolism control. Thus, STG could shape the gut microbiota, which has implications for the pathogenesis of T2DM. We hypothesized that the effects of STG on T2DM rats were partially associated with anti-inflammatory bacteria, whose metabolites could enhance epithelial barrier function, improve gastrointestinal health, inhibit inflammation, and ameliorate insulin resistance and attenuate T2DM. Based on the above findings, the modification of the gut microbiota structure and phylum composition might represent proposed mechanisms by which T2DM is improved by STG. STG could be used as a new CHF for managing diabetes. We suggest that STZ exerts systemic therapeutic effects through the gut microbiota, which might be helpful for clarifying its antidiabetic effects in vivo or in vitro and its downstream biological significance in further studies.

## Figures and Tables

**Table 1:** Relationship between common gut microbiota and diabetes mellitus.

Phylum	Genus	Tendency	Role in T2DM Risk Associated Factors
Firmicutes	*Allobaculum*	increased	Improve T2DM
	*Lactobacillus*	decreased	Aggravated T2DM
	*Ruminococcus*	decreased	Aggravated T2DM
	*Coprococcus*	decreased	Aggravated T2DM
	*Clostridium*	decreased	Aggravated T2DM
	*Lachnospira*	decreased	Aggravated T2DM
	*Coprobacillus*	Increased	Improve T2DM
	*Finegoldia*	Increased	Improve T2DM
	*Turicibacter*	Increased	Improve T2DM
	*Trichococcus*	Increased	Improve T2DM
	*Streptococcus*	Increased	Improve T2DM
	*Anaerostipes*	Increased	Improve T2DM
	*Staphylococcus*	Increased	Improve T2DM
	*Dorea*	Increased	Improve T2DM
	*Anaerofustis*	Increased	Improve T2DM

Bacteroidetes	*Akkermansia*	Increased	Improve T2DM
	*Adlercreutzia*	decrease	Improve T2DM
	*Alistipes*	decreased	Aggravated T2DM

Proteobacteria	*Desulfovibrionaceae*	decreased	Improve T2DM
	*Deltaproteobacteria *	increased	Aggravated T2DM
	*Desulfovibrionales*	increased	Aggravated T2DM
	*Psychrobacter*	Increased	Improve T2DM

## Data Availability

The data used to support the findings of this study are available from the corresponding author upon request when the research is accepted.
